# A cross-age odyssey of cognitive reading attributes: a scoping review

**DOI:** 10.3389/fpsyg.2025.1605898

**Published:** 2025-06-20

**Authors:** Muhamad Firdaus Mohd Noh, Mohd Effendi Ewan Mohd Matore, Nur Ainil Sulaiman

**Affiliations:** ^1^Faculty of Education, Universiti Kebangsaan Malaysia, Bangi, Selangor, Malaysia; ^2^Research Centre of Education Leadership and Policy, Faculty of Education, Universiti Kebangsaan Malaysia, Bangi, Selangor, Malaysia; ^3^Centre of Teaching and Learning Innovation, Faculty of Education, Universiti Kebangsaan Malaysia, Bangi, Selangor, Malaysia

**Keywords:** reading skills, cognitive reading attributes, language assessment, scoping review, cognitive diagnostic

## Abstract

Reading is a fundamental cognitive-linguistic process that involves the dynamic interaction of multiple interrelated cognitive and perceptual mechanisms. Existing reading models are often limited in fully capturing the intricate relationships between reading attributes across different age groups. This review aims to compare the cognitive attributes utilized in reading assessments for young and adult readers. Using a scoping review methodology, the study analyzed 47 empirical studies selected through a systematic search of reputable academic databases, Scopus, Web of Science, and Google Scholar, from an initial pool of 331 publications. The selection followed a screening process based on three inclusion and exclusion criteria: types of publications, language, and skills assessed. A distinctive pattern emerges in the assessment of cognitive reading skills across age groups, with adult readers evaluated on a wider array of attributes that encompass both fundamental skills and higher order cognitive abilities. In contrast, young readers' assessments tend to center on a narrower spectrum of subskills, primarily emphasizing literal and interpretive comprehension. This developmental pattern calls for the refinement of existing assessment models to better capture the progressive nature of cognitive reading development. Tailoring assessment tools and instructional strategies to align with learners' cognitive demands is imperative for internal stakeholders, while external stakeholders are urged to develop age-appropriate assessments. Future research could address the study's limitations by exploring advanced technologies, such as eye tracking, conducting rigorous reviews, performing cross-linguistic comparative studies, and evaluating diverse assessment methods to enhance the accuracy, effectiveness, and generalizability of reading assessments across various contexts.

## 1 Introduction

Reading is a fundamental cognitive-linguistic process that involves the dynamic interaction of multiple interrelated cognitive and perceptual mechanisms. As a core aspect of language comprehension, reading requires the coordination of lexical access, working memory, syntactic parsing, and semantic integration, enabling individuals to construct meaning from written texts (Hosseini Alast and Baleghizadeh, 2024). Unlike other polytomous language skills such as speaking and writing, which involve productive language use and require rater involvement for assessment (Mohd Noh and Mohd Matore, 2022, 2020), reading is a visually mediated process that necessitates efficient orthographic, phonological, and semantic activation to support fluent comprehension (Teng and Mizumoto, 2024). The psychological mechanisms underlying reading are deeply interconnected with broader cognitive functions, including executive control, attentional regulation, and memory retrieval, which contribute to individual differences in reading proficiency. Due to its complexity and the diverse theoretical perspectives that frame it, including developmental, cognitive, pedagogical, and computational frameworks, reading requires a comprehensive assessment approach capable of capturing its full range of cognitive attributes and skills. Within the psychology of language, multiple theoretical frameworks have been proposed to explain how cognitive and linguistic processes converge during reading. Classic reading models, such as psycholinguistic reading model (Goodman, 1967) and information processing model (Gough, 1972) provide insights into how readers derive meaning from texts. More recent perspectives such as the interactive-compensatory model (Stanovich, 1980). More recent models, like the cognitive processing model (Khalifa and Weir, 2009) and the direct and indirect effects model of reading (Kim, 2020), emphasize the hierarchical and interactive nature of reading, where multiple cognitive and contextual skills directly and indirectly contribute to comprehension.

While these models offer valuable insights into reading mechanisms, they often overlook developmental variations in cognitive reading processes. The way young and adult readers engage with text differs not only in terms of linguistic complexity but also in the cognitive operations employed during comprehension. Research has shown that adult readers rely more on higher-order cognitive functions, such as inference-making and semantic integration, while young readers focus more on basic decoding and literal comprehension (Sieo et al., 2022). However, existing reading models do not always explicitly address the gradual shifts in cognitive reading attributes throughout the lifespan. To address this gap, this review examines how cognitive reading attributes develop across different age groups by synthesizing findings from empirical studies. Another key limitation in traditional reading models is their reliance on broad theoretical constructs rather than fine-grained, diagnostic insights into individual cognitive mechanisms. Cognitive Diagnostic Assessment (CDA) emerges as an innovative framework capable of providing such granularity, offering a psychometric approach that systematically examines specific cognitive reading attributes. Distinct from conventional reading assessments, which yield general single proficiency scores, CDA enables a detailed diagnosis of strengths and weaknesses in core reading processes. This methodological advancement aligns with contemporary perspectives in reading cognition, developmental psycholinguistics, and the psychology of language by emphasizing a learner-specific approach to reading assessment. Despite its potential, research integrating CDA into developmental reading studies remains underexplored. While existing CDA studies have successfully retrofitted high-stakes assessments to diagnose reading attributes (Safari and Ahmadi, 2023), fewer studies have systematically applied CDA to trace the evolution of cognitive reading attributes across different age groups. Thus, the current review aims to identify and compare the reading attributes used in previous research to measure the reading skills of individuals from different age groups.

## 2 Literature review

### 2.1 Reading models

The diversity of reading models reflects various theoretical perspectives on how individuals engage with and comprehend texts. Table 1 presents the different models of reading (Goodman, 1967). Early conceptualization of reading as a psycholinguistic guessing game suggests that readers rely on contextual cues, linguistic structure, and prior knowledge to make predictions during reading, emphasizing the role of higher-level cognitive processes in guiding comprehension. This model exemplifies a top-down approach to reading, where meaning is primarily constructed through the reader's background knowledge and cognitive expectations rather than solely relying on the text itself. In contrast, Gough's (1972) and LaBerge and Samuels's (1974) stage-based models also known as the information processing model describe reading as a sequential process where readers decode text progressively, from letters to words to complete comprehension, highlighting the role of automaticity in word recognition and information processing. These models represent a bottom-up approach to reading, where comprehension is built from the accurate and systematic decoding of smaller units of text, such as letters and words before higher-level meaning can be constructed.

**Table 1:** Types of reading models and their characteristics.

**Type of reading models**	**Characteristics**
Psycholinguistic reading model (Goodman, 1967)	Reading is an active process where meaning is constructed from the overall context and prior knowledge, with the reader forming and testing hypotheses about the text.
Information processing model (Gough, 1972)	Reading involves sequential processing from recognizing individual letters to understanding the entire text, with all steps working together for comprehension.
Automatic processing model (LaBerge and Samuels, 1974)	Reading is an automatic process where word stimuli are transformed into meaning through several memory systems.
Three-component reading model or socio-cultural reading model (Coady, 1979)	Reading comprehension is influenced by cognitive skills and sociocultural factors, depending on conceptual ability, background knowledge, and processing strategies.
Two-component reading model or simple view of reading model (SVR; Hoover and Gough, 1990)	Reading comprehension results from the development of word identification and linguistic comprehension over time.
Interactive reading model (Rumelhart, 1977)	Reading is a dynamic interaction between prior knowledge and the text, with top-down and bottom-up processes occurring simultaneously and relying on mental schemas.
Interactive-compensatory reading model (Stanovich, 1980)	Reading involves compensating for weaknesses in one area with strengths in another, using both bottom-up and top-down processes to construct meaning.

Apart from these stage-based reading models, component-based models, such as the sociocultural reading model, describe how a reader's background knowledge, conceptual abilities, and process strategies work together to create comprehension (Coady, 1979). Similarly, the Simple View of Reading conceptualizes reading comprehension as the product of two core components: decoding and linguistic comprehension, emphasizing that both are necessary for proficient reading (Hoover and Gough, 1990). This model highlights that weaknesses in either decoding or comprehension can hinder overall reading ability, underscoring the importance of balanced instruction targeting both skills. Meanwhile, the interactive reading model integrates both top-down and bottom-up processes, emphasizing a compensatory mechanism where strengths in one cognitive process balance weaknesses in another (Stanovich, 1980; Rumelhart, 1977). Rumelhart's (1977) interactive model synthesizes these perspectives by proposing that reading involves a dynamic interaction of both text-driven (perceptual) and knowledge-driven (cognitive) processes occurring simultaneously. Whereas, Stanovich's (1980) interactive-compensatory model extends the idea by suggesting that strengths in one cognitive process, such as contextual knowledge, can offset weaknesses in another, such as phonological decoding.

Expanding on these foundational models, the cognitive processing model of reading (Khalifa and Weir, 2009) integrates cognitive psychology perspectives to describe reading as a dynamic process involving both bottomup and top-down processing. It emphasizes the importance of goal setting during reading and distinguishes between local comprehension (understanding specific details) and global comprehension (grasping overall meaning), illustrating the complex interplay of cognitive skills required for effective reading. Similarly, the integrated reading model, also referred to as the Direct and Indirect Effects Model of Reading (DIER; Kim, 2020) proposes reading comprehension as an outcome of hierarchical, dynamic, and interactive contributions from multiple language, cognitive, and contextual skills. This model explains how some skills, such as word recognition, directly impact comprehension, while others, such as working memory and inferencing, provide indirect support by facilitating the integration of information. These models collectively aim to explain the complex, multi-stage nature of reading, emphasizing the interaction of perceptual, cognitive, and contextual mechanisms rather than rigid classifications, while acknowledging the dynamic and adaptive nature of reading comprehension.

### 2.2 Reading taxonomies and cognitive structures

Reading taxonomies provide systematic classification framework that organizes and categorizes the various cognitive skills, processes, and components involved in reading comprehension. These reading taxonomies underscore the varied approaches to categorizing and assessing the intricate skills involved in reading, distinguishing them from reading models, which focus on the theoretical frameworks of the reading process itself. Each taxonomy emphasizes different aspects of reading comprehension and cognitive processes. Reading taxonomy by Davis (1968) focuses on sub-skills like recalling word meanings, making inferences, and understanding the author's technique, which was empirically tested on students. Munby's (1978) taxonomy, often used in curriculum development, outlines 19 sub-skills, including identifying main ideas and interpreting text through various cohesion devices, though it has been critiqued for lacking empirical validation. Heaton's (1991) taxonomy provides a detailed list of 14 sub-skills, such as deducing word meaning and understanding conceptual meaning, aimed at assessing specific reading skills. Meanwhile, Hughes and Hughes's (2020) divide reading skills into expedient and careful reading, further categorizing them into sub-skills like identifying discourse markers and interpreting complex sentences. Whereas, Anderson and Krathwohl (2001) updated version of Bloom's Taxonomy introduces a cognitive dimension to reading by classifying skills into six levels: remembering, understanding, applying, analyzing, evaluating, and creating. This hierarchical model is echoed in Luebke and Lorié (2013) divides reading skills into four sub-skills: identification, understanding and analyzing, making inferences, and application. Each of these taxonomies provides a different lens through which reading comprehension can be assessed, from the recall of explicit information to higher-order cognitive skills such as critical thinking and application. Collectively, these taxonomies highlight the complexity of reading comprehension and offer valuable tools for educators and researchers to assess and enhance reading skills in a structured and systematic manner.

### 2.3 The exploration of reading attributes through cognitive diagnostic assessment

An attribute refers to a specific cognitive skill or ability that contributes to task performance, particularly in educational contexts (Wang et al., 2021). Cognitive attributes are the foundational components underlying a learner's ability to process, interpret, and respond to tasks, making them essential units for understanding academic performance (Zhang et al., 2024). Meanwhile, cognitive reading attributes are the cognitive skills involved in the process of reading and comprehending text. These attributes encompass a range of cognitive processes, including word recognition, decoding, inferencing, summarizing, comprehension monitoring, and understanding text structures (Li et al., 2021). Together, they influence a reader's ability to extract, interpret, and construct meaning from written material. Understanding these attributes is crucial for assessing reading proficiency and guiding instructional strategies tailored to individual learner needs. The underlying sub-skills of reading have been explored in previous research studies using various approaches, including factor analysis (Nightingale et al., 2023; Lin, 2024), structural equation modeling (SEM) (Lee and Lee, 2023; Peng et al., 2021), item response theory (IRT) (Geramipour et al., 2021; Polat et al., 2022), and cognitive diagnostic modeling (CDM) (Askari and Karami, 2024; Chen et al., 2023), each providing unique insights into the cognitive processes and relationships among reading components.

Cognitive reading attributes have been investigated within CDA principles by examining how specific cognitive skills contribute to reading comprehension and how they can be accurately measured (Mei and Chen, 2022). CDA represents a modern advancement in educational measurement by integrating psychological and measurement theories that offers insights into students' cognitive processes and addresses the limitations of conventional assessment methods (Maas et al., 2024). CDA provides a holistic and contextual understanding of individual cognitive processes by diagnosing specific cognitive reading attributes, drawing on insights from psychological theories and advanced measurement techniques (Ketabi et al., 2021). Other approaches often provide a narrow view of students' cognitive capabilities, falling short to diagnostically capture the intricacies of reading comprehension and the diverse cognitive reading attributes involved (von Davier and Lee, 2019).

The evolution of CDA is deeply rooted in the historical development of educational psychology and measurement theories. Early educational psychology prioritized intelligence measurement through specific tests, focusing on intellectual and academic skills (van der Linden, 2016). However, as understanding of human cognition expanded, theories such as cognitive development theory (Piaget, 1950) and information processing theory (Miller, 1956) highlighted the importance of cognitive processes like information handling, storage, and retrieval—key elements in reading comprehension. Concurrent advancements in measurement theories supported a more holistic and contextual approach to assessing these cognitive processes (Miller and Lovler, 2020). This integration ultimately led to the development of CDA models, which enable the exploration of cognitive reading attributes across various contexts, including language skills (Sessoms and Henson, 2018).

One common method used within CDA studies for investigating reading attributes is the retrofitting of existing high-stakes assessments for diagnostic purposes. Retrofitting involves reanalyzing existing reading tests to ensure they provide relevant and useful diagnostic information (Sessoms and Henson, 2018). This approach has been applied to large-scale assessments such as the Program for International Student Assessment (PISA; Chen and Chen, 2016) and the Test of English as a Foreign Language (TOEFL; Safari and Ahmadi, 2023), among others. Retrofitting allows educators to gain diagnostic insights from assessments not originally designed for this purpose, although challenges remain, such as ensuring a sufficient number of items to measure specific cognitive reading attributes accurately (Gierl and Cui, 2008). Despite the efficiencies of retrofitting, some researchers advocate for the development of entirely new CDA items and assessments tailored specifically to measure reading attributes. Although more resource intensive, this approach allows for a more detailed and personalized analysis of learners' cognitive strengths and weaknesses in reading. For example, a study in Turkey developed a diagnostic reading test for university students (Toprak and Cakir, 2021). Developing new CDA items ensures that the assessments are specifically designed to measure key cognitive reading attributes, such as decoding, inferencing, and comprehension, providing more precise diagnostic information and supporting targeted instructional interventions (Ketabi et al., 2021).

## 3 Methods

### 3.1 Design

The current study employed a scoping review method as it is appropriate for gaining an in-depth understanding of a broad issue and charting existing research to better inform future studies (Munn et al., 2022). The key difference between a scoping review and a systematic literature review is that while a systematic review focuses on synthesizing specific evidence to answer a well-defined research question (Sabtu and Mohd Matore, 2024), a scoping review aims to map the existing literature on a broader topic, identifying gaps and trends without necessarily assessing the quality of individual studies (Nadmilail et al., 2023). It is particularly useful for analyzing emerging evidence when it is uncertain if more specific questions can be effectively addressed by a precise systematic review (Pollock et al., 2021). Scoping reviews are ideal for evaluating and understanding the extent of knowledge in a developing field, as well as for identifying, mapping, reporting, or discussing the characteristics and concepts within that area (Peters et al., 2022). The scoping review was conducted based on the guidelines recommended by Levac et al. (2010) and Arksey and O'Malley (2005) which involves five stages: (i) identifying the research objectives, (ii) identifying relevant studies, (iii) selecting the studies, (iv) charting the data, and (v) collating, summarizing, and reporting the results.

### 3.2 Review protocol

The review was guided by the Preferred Reporting Items for Systematic reviews and Meta-Analyses extension for Scoping Reviews (PRISMA-ScR). The review process included the identification of relevant studies, their selection based on predefined criteria, and the abstraction and synthesis of key data. All screening and selection processes were conducted independently by two reviewers, following the PRISMA-ScR guidelines to minimize bias and ensure the reliability of the findings.

This rigorous process ensures the inclusion of high-quality evidence to address the research questions posed in this study.

### 3.3 Research question formulation

The research questions were formulated based on the Population, Interest and Context (PICo) framework (Lockwood et al., 2015). The PICo framework aids researchers in crafting appropriate research questions for reviews by focusing on the core elements (Mohamed Shaffril et al., 2021). The current review has applied these components by examining, (i) Population: readers across different age groups, (ii) Interest: cognitive reading attributes, and (iii) Context: cognitive diagnostic assessment studies. As a result, the review is guided by three research questions:

1. What are the publication trends in cognitive diagnostic assessment studies on reading, specifically in relation to test types, sample demographics and age distributions?
2. How do cognitive diagnostic assessment studies conceptualize and operationalize key reading attributes for young and adult readers?
3. How do the similarities and differences in cognitive reading attributes assessed in young and adult readers reflect their cognitive and linguistic development?

### 3.4 Database

The review process began with the development of a systematic search strategy. This involved searching through three reputable academic databases, Scopus, Web of Science and Google Scholar. These databases have been used by previous researchers in reviewing the latest publication trends in various fields, providing wide coverage of peer-reviewed journals, conference papers, and other scholarly works (Sakaria et al., 2023). These databases were selected due to their extensive reach and credibility, ensuring that the review captures a broad spectrum of relevant and up-to-date research findings (Sabtu and Mohd Matore, 2024). Scopus, an esteemed database, aggregates content from over 30,000 journals representing a wide range of subject fields, including education, measurement, and language, enabling researchers to identify trends, gaps, and advancements in the field of study (Masdoki et al., 2021). This database, encompassing contributions from more than 11,000 publishers, undergoes a rigorous peer-review process. On the other hand, the Web of Science (WoS) is a platform providing access to a robust database across numerous academic disciplines (Singh et al., 2021). Originally established by the Institute for Scientific Information (ISI) and currently monitored by Clarivate Analytics, WoS covers over 30,000 journals spanning more than 250 disciplines (Birkle et al., 2020). Meanwhile, Google Scholar provides extensive access to a wide range of academic literature, including peer-reviewed articles, theses, books, and conference papers across various disciplines (Martín-Martín et al., 2021). Additionally, Google Scholar's extensive indexing of both traditional and gray literature ensures a thorough and inclusive search of relevant studies.

### 3.5 Systematic search strategies

**Figure 1:**
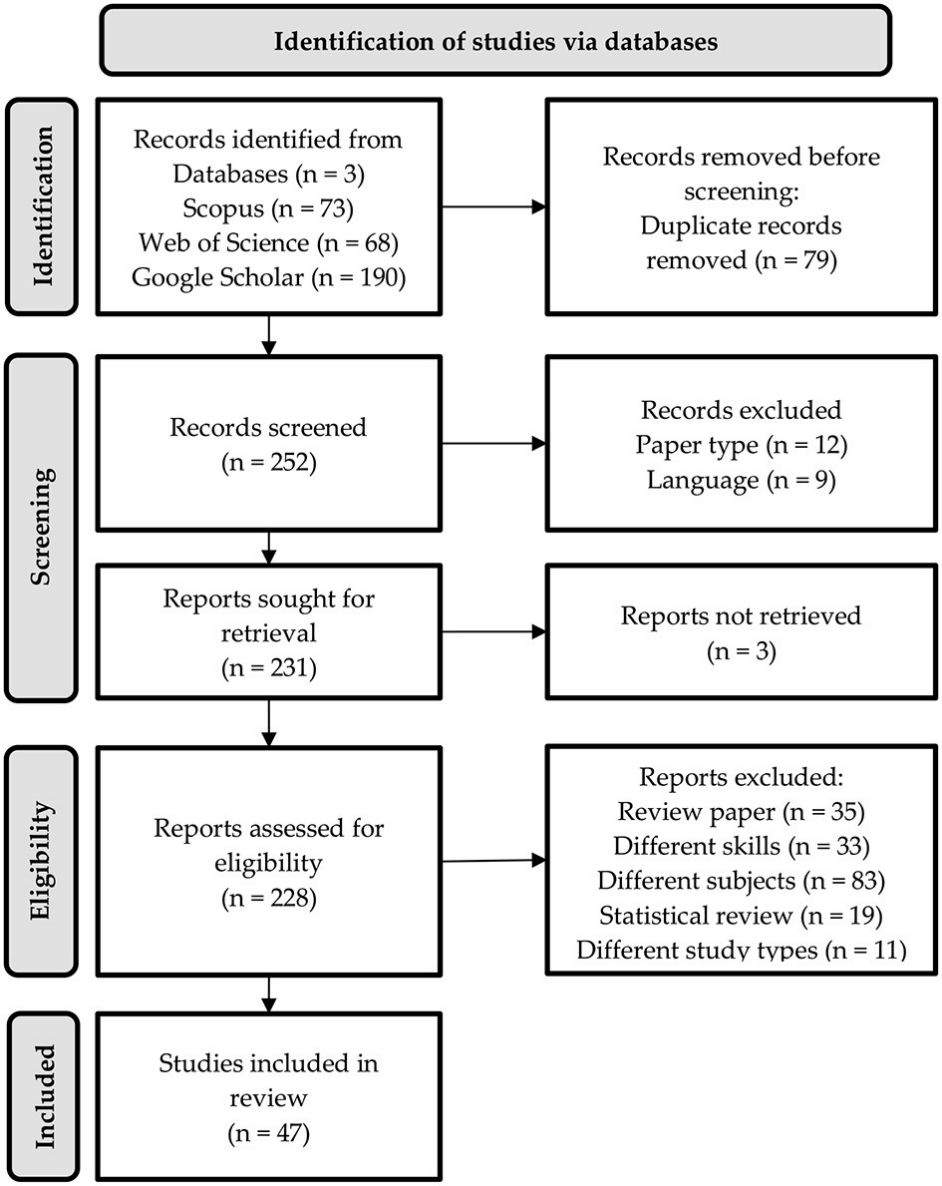
PRISMA flowchart of the study.

This stage involves three steps; identification, screening, and eligibility, as illustrated in Figure 1.

#### 3.5.1 Identification

The identification process begins with selecting appropriate keywords. To maximize the retrieval of relevant articles for the current review, the keywords are categorized into two groups: reading assessment and cognitive diagnostic assessment. Related terms for each keyword were identified and shown in Table 2. By combining the keywords and related terms, the search process was conducted on the three databases with respective search strings as displayed in Table 3. The search on Scopus and Web of Science used a structured string incorporating Boolean operators, proximity operators, and wildcard symbols ^*^ to ensure relevant results. Boolean operators (OR) broadened the search by including synonyms like “reading assess^*^” OR “reading test^*^” OR “reading evaluat^*^”. Proximity operators (W/) refined the results by ensuring terms like “assess^*^ W/reading” and “test^*^ W/reading” appeared close together, maintaining relevance. Wildcards (^*^) captured word variations, such as “assess^*^” for “assess”, “assessment”, or “assessed”. Keywords like “cognitive diagnos^*^” and “classification AND diagnos^*^” ensured a focus on cognitive and diagnostic aspects. This strategy effectively targeted studies on reading assessment and cognitive diagnostics. Whereas, the search of the Google Scholar database was conducted using the Publish or Perish version 8.17 for Windows software, a tool designed to retrieve and analyze academic citations. This software allowed for efficient querying of Google Scholar using the same structured keywords and phrases as the Scopus and Web of Science searches. The search yielded a total of 331 publications, with 73 articles retrieved from Scopus, 68 from Web of Science, and 190 from Google Scholar.

**Table 2:** Keywords and related terms.

**Keywords**	**Related terms**
Reading assessment	Reading skill assessment, assessing reading, reading test, testing reading, evaluating reading
Cognitive diagnostic assessment	Cognitive diagnostic approach, cognitive diagnostic model, diagnostic classification model

**Table 3:** Search string.

**Databases**	**Search strings**
Scopus	((“reading assess^*^” OR “assess^*^ W/reading” OR “reading test^*^” OR “test^*^ W/reading” OR “reading evaluat^*^” OR “evaluat^*^ W/reading” OR “reading comprehension” OR “skill-based assessment” OR “language assessment” OR “language testing”) AND (“cognitive diagnos^*^” OR “classification AND diagnos^*^”))
Web of Science	((“reading assess^*^” OR “assess^*^ W/reading” OR “reading test^*^” OR “test^*^ W/reading” OR “reading evaluat^*^” OR “evaluat^*^ W/reading” OR “reading comprehension” OR “skill-based assessment” OR “language assessment” OR “language testing”) AND (“cognitive diagnos^*^” OR “classification AND diagnos^*^”))
Google Scholar	“cognitive diagnos^*^” AND “reading skill”

#### 3.5.2 Screening

Following the identification stage, 78 duplicate records were removed, leaving 253 records for initial screening. During the screening stage, the records were assessed for relevance based on predefined inclusion and exclusion criteria as presented in Table 4. To ensure transparency and reduce the risk of selection bias, two independent reviewers conducted the title and abstract screening. Discrepancies between the reviewers were resolved through discussion until consensus was reached. If disagreements persisted, a third reviewer was consulted. The first criterion was the types of publications. Only publications on empirical studies were selected but not empirical studies using simulation data and other types of studies, including conceptual and review studies. The second criterion applied was language. Only publications in the English language were accepted. As a result, 21 records were excluded, with 12 records removed due to inappropriate paper types such as conceptual papers and book chapters, while nine records were excluded due to language limitations such as Chinese language and Persian language. This reduced the number of reports to 232, which were then sought for retrieval. However, three reports could not be retrieved, leaving a total of 229 reports for further evaluation.

**Table 4:** Screening criteria.

**No**.	**Criteria**	**Acceptance criteria**	**Rejection criteria**
1.	Types of publication	Empirical studies with real data	Other than empirical studies with real data—empirical studies using simulation data, conceptual papers, review papers, method reviews
2.	Languages	English language	Other than English—publications reported in other than English language, studies on assessment of other than English language
3.	Focused skills	Reading skills	Other than reading skills—writing, speaking, listening skills

#### 3.5.3 Eligibility

In the eligibility stage, the remaining reports were assessed in detail to determine their suitability for the review. Again, two reviewers independently assessed the full-text articles using the predefined inclusion and exclusion criteria. This dual-reviewer approach enhanced the methodological rigor of the review process. A total of 181 reports were excluded for various reasons: 35 were review papers, 33 focused on different skills, 83 addressed subjects outside the scope of the review such as Mathematics, diagnostic assessment and computerized adaptive testing (CAT), 19 were statistical reviews, and 11 followed different study types that did not meet the inclusion criteria, such as action research. Following this rigorous screening and eligibility process, 47 studies were deemed relevant and included in the final review. This multi-step process ensured the inclusion of only the most pertinent and high-quality studies, allowing for a robust and reliable scoping review of cognitive reading attributes across different age groups. The next step involved graphically presenting the selected articles. Summaries were created for each article, detailing the author, year, title of the study, methodologies, population, samples and test names. The final phase of this review framework included compiling, summarizing, and reporting the results. All data were stored and processed using Mendeley Desktop Version 1.19.8. A synthesis of the literature was then created by summarizing the key points and presenting them in text, tables, and figures. All 47 publications were thoroughly retrieved and reviewed in their entirety to ensure they met the inclusion criteria.

### 3.6 Systematic coding

Each publication was coded based on six variables: study design, test names, samples, sample types, sample ages, and cognitive attributes. Table 5 outlines the definitions and possible codes for each variable. The study design refers to the scientific framework of the study, categorized as either a retrofitting study or a true study. Test names indicate the reading assessments used, including wellknown standardized tests such as the Progress in International Reading Literacy Study (PIRLS) and the Program for International Student Assessment (PISA), as well as newly developed reading tests. The samples represent the test-takers, which include primary school students, high school students, undergraduate students, and postgraduate students. Sample types further classify test-takers based on their reading development stage, distinguishing between young and adult readers. Meanwhile, sample age specifies whether the exact age of the participants is mentioned or not. Lastly, cognitive attributes refer to the specific reading skills assessed in each study, such as finding explicit information (EXP), making inferences (INF), generalizing or synthesizing main ideas (GEN), interpreting texts (INT), lexical or vocabulary knowledge (LEX), and evaluating or analyzing texts (EVA). By coding publications according to these variables, the review systematically organizes reading research, allowing for a structured analysis of trends, patterns, and gaps in the field. This approach enhances the understanding of how reading skills have been assessed across various studies and contexts. The preparation of data for presentation and synthesis, including handling missing summary statistics, data conversions, and visual display of individual study results, was conducted using Microsoft Excel 2021.

**Table 5:** Article coding for the present review.

**No**.	**Variable**	**Definition**	**Possible codes**
1.	Study design	Scientific design of the study	Retrofitting study, true study
2.	Test names	The name of the test where the data was analyzed or the name of the newly developed test	Progress in International Reading Literacy Study (PIRLS), Program for International Student Assessment (PISA), Grade 6 provincial reading achievement assessment, newly developed reading test
3.	Samples	The test-takers	Primary school students, high school students, undergraduate students and postgraduate students
4.	Sample types	The classification of the samples based on the purpose of the test	Young reader, adult reader
5.	Sample age	The age of the test-takers	Exact age of the samples, age range, not mentioned
6.	Attributes	The sub-skills used or analyzed to assess reading skills of the study samples	Finding explicit information (EXP), making inferences (INF), generalizing or synthesizing main ideas (GEN), making interpretation of texts (INT), lexical or vocabulary knowledge (LEX), evaluating or analyzing the text (EVA)

## 4 Results

### 4.1 RQ1: What are the publication trends in cognitive diagnostic assessment studies on reading, specifically in relation to test types, sample demographics and age distributions?

**Figure 2:**
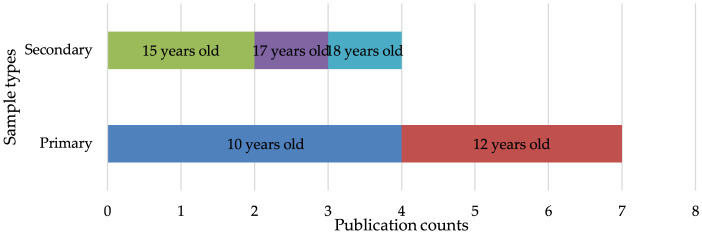
Publication counts based on sample types and ages.

The analysis identified 11 articles focusing on young readers, with each study utilized different types of reading tests. Table 6 shows the reading tests and sample profiles used in the studies. Only one study developed new reading assessment items (Nallasamy and Khairani, 2022), while the other 10 studies retrofitted existing non-diagnostic reading assessment items to extract the diagnostic reports such as from a large-scale K−12 ELP assessment measuring the social and academic language (Yumsek, 2023), Progress in International Reading Literacy Study (PIRLS; Toprak-yildiz, 2021), Program for International Student Assessment (PISA) (Chen and Chen, 2016), Grade 6 provincial reading achievement assessment (Jang et al., 2013, 2015), Grade 5 and 6 reading comprehension test (Liu and Bian, 2021), and the National Matriculation English Test (NMET) (Fan and Yan, 2020). In regards to the types of the samples, seven of these studies focused on primary school students, while the remaining four targeted secondary school students, as shown in Figure 2. Among the primary school samples, four studies involved students aged ten (Nallasamy and Khairani, 2022; Thi and Loye, 2019), and three studies involved students aged 12 (Jang et al., 2013, 2015). Regarding the secondary school samples, two studies focused on students aged 15 (Chen and Chen, 2016, 2015), while one study each involved students aged 17 (Fan and Yan, 2020) and 18 (Yumsek, 2023), respectively.

**Table 6:** Reading tests and sample profiles in CDA studies involving young readers.

**No**.	**Study**	**Reading tests**	**Sample types**	**Sample ages**
1.	Yumsek (2023)	A large-scale K−12 ELP assessment	Secondary school students	17 years old
2.	Nallasamy and Khairani (2022)	A newly-developed cognitive diagnostic reading test	Primary school students	10 years old
3.	Toprak-yildiz (2021)	Progress in International Reading Literacy Study (PIRLS)	Primary school students	10 years old
4.	George and Robitzsch (2021)	Progress in International Reading Literacy Study (PIRLS)	Primary school students	10 years old
5.	Liu and Bian (2021)	A large-scale Grade 5 and 6 reading comprehension test	Primary school students	11–12 years old
6.	Fan and Yan (2020)	National Matriculation English Test (NMET)	Secondary school students	18 years old
7.	Thi and Loye (2019)	Progress in International Reading Literacy Study (PIRLS)	Primary school students	10 years old
8.	Chen and Chen (2016)	Program for International Student Assessment (PISA)	Secondary school students	15 years old
9.	Chen and Chen (2015)	Program for International Student Assessment (PISA)	Secondary school students	15 years old
10.	Jang et al. (2015)	Grade 6 provincial reading achievement assessment	Primary school students	12 years old
11.	Jang et al. (2013)	Grade 6 provincial reading achievement assessment	Primary school students	12 years old

A total of 36 articles sampling adult readers were further analyzed after the eligibility procedure. The reviewed studies employed a variety of reading tests and sample groups. Table 7 depicts the names of the reading tests, the types and the age of the samples. Findings reveal that only five studies developed new reading assessment items (Askari and Karami, 2024; Toprak and Cakir, 2021; Alavi and Ranjbaran, 2018; Liu and Read, 2023; Toprak-Yildiz, 2022). In contrast, the remaining 31 studies employed a retrofitting approach by adapting existing tests to analyze cognitive attributes of reading skills and to produce diagnostic profiling reports using different types of tests such as high-stakes language proficiency test (Boori et al., 2023), test for English Majors (Chen et al., 2023), International English Language Testing System (IELTS) (Jang et al., 2023) and high-stakes university entrance examination (Tonekaboni et al., 2021). Meanwhile, the study samples represent five distinct groups: undergraduate students, postgraduate students, undergraduate student candidates, postgraduate student candidates, and specific international examination test takers. While the majority of the studies did not report the ages of the samples, 12 studies did provide age ranges. None of these studies focused on a single age group; instead, the samples encompassed various age ranges. The youngest participants were 18 years old (Toprak-Yildiz, 2022) and the oldest was 59 years old (Jang et al., 2023).

**Table 7:** Reading tests and sample profiles in CDA studies involving adult readers.

**No**.	**Study**	**Reading test**	**Sample types**	**Sample ages**
1.	Askari and Karami (2024)	Newly developed	Bachelor's and master's degrees students	19–47 years old
2.	Boori et al. (2024)	Iranian high-stakes language proficiency test	PhD students	Not mentioned
3.	Boori et al. (2023)	Iranian high-stakes language proficiency test	PhD students	Not mentioned
4.	Chen et al. (2023)	Test for English Majors (TEM)	Final year bachelor's degree students	Not mentioned
5.	Jang et al. (2023)	International English Language Testing System (IELTS)	IELTS test-takers	14–59 years old
6.	Mohammed et al. (2023)	B1 Preliminary English Test (PET)	Tertiary education students	19 and 39 years old
7.	Shahmirzadi and Marashi (2023)	General English test	PhD students	Not mentioned
8.	Sun and Hwang (2023)	College English Test Band Four (CET-4)	Tertiary education students	Not mentioned
9.	Wang (2023)	Large-scale Spanish proficiency test	Bachelor's degree students	21–22 years old
10.	Cai and Chen (2022)	Test for English Majors Band 4 (TEM-4)	Bachelor's degree students	Not mentioned
11.	He et al. (2022)	Large scale in-house EFL exit test	Bachelor's degree students	19–21 years old
12.	Toprak-Yildiz (2022)	Newly developed	Bachelor's degree students	18–20 years old
13.	Du and Ma (2021)	College English Test Band 4 (CET-4)	First year bachelor's degree students	Not mentioned
14	Tonekaboni et al. (2021)	High-stakes university entrance examination (UEE) Master of Arts (M.A.) exam	Master degree's students	Not mentioned
15	Toprak and Cakir (2021)	A new diagnostic L2 reading comprehension test	Bachelor's degree students	18–23 years old
16	Liu and Read (2021)	DELNA reading assessment	Bachelor's degree students	Not mentioned
17	Hemati and Baghaei (2020)	Iranian University Entrance Examination (IUEE)	Bachelor's degree candidates	Not mentioned
18	Mirzaei et al. (2020)	International English Language Testing System (IELTS)	IELTS test-takers	Not mentioned
19	Tabatabaee-Yazdi (2020)	Iranian University Entrance Examination (IUEE)	Bachelor's degree candidates	20–44 years old
20	Zhao et al. (2020)	University final English examination	First year bachelor's degree students	Not mentioned
21	Javidanmehr et al. (2019)	University entrance exam for PhD programs	PhD candidates	25–50 years old
22	Ravand (2019)	Iranian University Entrance Examination (IUEE)	Master's degree candidates	Not mentioned
23	Alavi and Ranjbaran (2018)	Newly developed Iranian National University	Bachelor's degree students	Not mentioned
24	Ravand and Robitzsch (2018)	Entrance Examination (INUEE) - master program candidate admission	Master's degree candidates	22–35 years old
25	Li and Wang (2017)	Test of Practical Chinese	Tertiary education students	Not mentioned
26	Ranjbaran and Alavi (2017)	Reading comprehension test for Bachelor's Degree General English course	Bachelor's degree students	Not mentioned
27	Ravand (2016)	Iranian National University Entrance Examination (INUEE)—master program candidate admission	Master's degree candidates	22–25 years old
28	Sook Yi (2016)	Examination for the Certificate of Proficiency in English (ECPE)	TOEFL test-takers	Not mentioned
29	Baghaei and Ravand (2015)	Iranian National University Entrance Examination (INUEE)	Master degree candidates	Not mentioned
30	Kim (2015)	Reading section of a placement test used in the adult ESL program	Tertiary education students	18 years old and above
31	Li et al. (2015)	Michigan English Language Assessment Battery (MELAB)	Tertiary education students	Not mentioned
32	Li and Suen (2013)	Michigan English Language Assessment Battery (MELAB)	Tertiary education students	Not mentioned
33	Jang (2009a)	LanguEdge Courseware reading test	Bachelor's degree students	Not mentioned
34	Jang (2009b)	LanguEdge Courseware reading test	Bachelor's degree students	Not mentioned
35	Lee and Sawaki (2009)	Test of English as a Foreign Language (TOEFL iBT)	TOEFL test-takers	Not mentioned
36	Sawaki et al. (2009)	Test of English as a Foreign Language (TOEFL iBT)	TOEFL test-takers	Not mentioned

**Figure 3:**
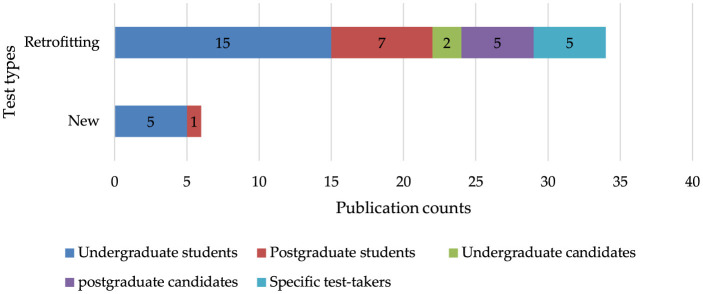
Publication counts based on test types and samples.

Figure 3 displays the publication counts categorized by test types and sample types. Among the five studies that developed new assessment items, all utilized undergraduate students (Toprak and Cakir, 2021; Alavi and Ranjbaran, 2018), with one study also combining undergraduate and postgraduate students (Askari and Karami, 2024). This kind of study, also known as true CDA studies, undergoes a rigorous process of designing cognitive models, constructing Qmatrix, developing and validating new items and finally profiling test-takers mastery classes. For retrofitting studies that adapted existing tests, 15 studies involved undergraduate students (Chen et al., 2023), seven involved postgraduate students (Boori et al., 2024), two focused on undergraduate student candidates (Hemati and Baghaei, 2020) and, respectively, five studies targeted postgraduate student candidates (Javidanmehr et al., 2019) and specific examination test takers (Jang et al., 2023). Retrofitting is ubiquitous in CDA studies due to the need to adapt existing assessments to align with cognitive diagnostic models, allowing researchers to extract detailed information about the test-takers strengths and weaknesses without developing entirely new tests (Mohd Noh and Mohd Matore, 2024).

### 4.2 RQ2: How do cognitive diagnostic assessment studies conceptualize and operationalize key reading attributes for young and adult readers?

The analysis of CDA studies reveals distinct approaches to defining and measuring reading attributes for both young and adult readers. Across the reviewed studies, reading attributes are conceptualized based on cognitive processing theories and language comprehension models. Based on the coding process, 13 codes of cognitive reading attributes emerged with at least one occurrence. Table 8 highlights the essential reading attributes employed in CDA studies to evaluate the reading skills of young readers. Topping the list are finding explicit information (EXP) and making inferences (INF). These are followed by generalizing or synthesizing main ideas (GEN), making interpretation of texts (INT), lexical or vocabulary knowledge (LEX), evaluating or analyzing the text (EVA), grammatical knowledge (GRAM), syntactic knowledge (SYN) and summarizing (SUM). Finally, others represent four other attributes with <3 occurrences, including sequencing, understanding charts, expressing in written forms and recognizing authors' styles.

**Table 8:** Key reading attributes of young readers in CDA studies.

**No**.	**Study**	**No. of Attributes**	**EXP**	**INF**	**GEN**	**INT**	**LEX**	**EVA**	**GRA**	**SYN**	**SUM**	**Others**
1.	Yumsek (2023)	6	/	/			/		/		/	/
2.	Nallasamy and Khairani (2022)	5	/	/	/		/			/		
3.	Toprak-yildiz (2021)	4	/	/		/		/				
4.	George and Robitzsch (2021)	9	/	/	/			/				/
5.	Liu and Bian (2021)	4	/	/	/							
6.	Fan and Yan (2020)	7	/	/	/		/		/	/		/
7.	Thi and Loye (2019)	5	/	/		/		/				/
8.	Chen and Chen (2016)	6	/	/	/	/		/				
9.	Chen and Chen (2015)	6	/	/	/	/		/				
10.	Jang et al. (2015)	4	/	/		/	/		/		/	
11.	Jang et al. (2013)	3	/	/		/	/		/		/	
Total			11	11	6	6	5	5	4	2	3	4

EXP, Finding explicit information; INF, Making inferences; GEN, Generalizing or synthesizing ideas; INT, Making interpretation; LEX, Lexical knowledge; EVA, Evaluating text; GRAM, Grammar knowledge; SYN, Syntactic knowledge; SUM, Summarizing ideas.

Table 9 presents key reading attributes of adult readers assessed in CDA studies. Regarding the number of attributes used to assess reading skills among adult readers, they ranged between 3 (Jang et al., 2023) and 10 (Kim, 2015). The coding process has produced 21 codes with at least one occurrence of attributes used in the reviewed studies to measure the reading skills of adult readers. The key attributes are led by lexical knowledge (LEX), making inferences (INF) and finding explicit information (EXP). These are followed by syntactic knowledge (SYN), summarizing ideas (SUM), grammar knowledge (GRAM), generalizing or synthesizing (GEN), identifying main ideas (MI), making interpretation (INT), connecting ideas (CON), recognizing authors' styles (AUT), scanning or skimming (SCAN), understanding paragraphs (PARA), evaluating (EVA), building a mental model (BUI), identifying supporting details (DET). Others refer to other attributes with <3 occurrences including negation, applying background knowledge, predicting, pragmatic knowledge and holding memory.

**Table 9:** Key reading attributes of adult readers assessed in CDA studies.

**Study**	**No of attributes**	**LEX**	**INF**	**EXP**	**SYN**	**SUM**	**GRAM**	**GEN**	**MI**	**INT**	**CON**	**AUTH**	**SCAN/SKM**	**PARA**	**EVA**	**BUILD**	**DET**	**Others**
Askari and Karami (2024)	7	/	/		/	/	/							/				
Boori et al. (2023)	5	/	/	/			/		/									
Boori et al. (2024)	5	/	/	/			/		/									
Chen et al. (2023)	8	/	/	/					/	/		/			/			
Jang et al. (2023)	3		/	/		/												
Mohammed et al. (2023)	4		/				/		/								/	/
Shahmirzadi and Marashi (2023)	5	/	/	/	/						/							
Sun and Hwang (2023)	6	/		/	/	/		/										
Wang (2023)	5	/		/	/	/												
Cai and Chen (2022)	5		/	/		/				/					/			
He et al. (2022)	4	/	/	/	/			/										
Toprak-Yildiz (2022)	5	/	/	/			/				/							
Du and Ma (2021)	8	/	/	/	/	/								/				
Tonekaboni et al. (2021)	6	/	/		/			/	/							/		
Toprak and Cakir (2021)	5		/	/	/							/		/				
Liu and Read (2021)	6	/	/	/		/		/		/								
Hemati and Baghaei (2020)	5	/	/	/			/								/			
Mirzaei et al. (2020)	6	/	/				/	/					/					
Tabatabaee-Yazdi (2020)	5	/	/	/			/								/			
Zhao et al. (2020)	5		/	/				/		/	/							/
Javidanmehr et al. (2019)	5	/	/	/	/						/							
Ravand (2019)	5	/	/			/	/							/				
Alavi and Ranjbaran (2018)	9	/	/	/		/				/		/	/					/
Ravand and Robitzsch (2018)	5	/	/		/				/								/	/
Li and Wang (2017)	8	/	/	/					/				/					/
Ranjbaran and Alavi (2017)	9	/	/	/		/				/		/	/					/
Ravand (2016)	5	/	/		/				/								/	/
Sook Yi (2016)	4	/		/				/			/							
Baghaei and Ravand (2015)	5	/	/		/				/								/	
Kim (2015)	10	/	/	/	/	/	/						/	/				/
Li et al. (2015)	4	/	/	/	/													
Li and Suen (2013)	4	/		/	/													
Jang (2009a)	9	/	/	/	/	/				/						/		/
Jang (2009b)	9	/	/	/	/	/				/						/		/
Lee and Sawaki (2009)	4	/		/				/										
Sawaki et al. (2009)	4	/		/				/				/						
Total		32	30	28	17	13	10	9	9	8	5	5	5	5	4	3	4	10

LEX, Lexical knowledge; INF, Making inferences; EXPL, Finding explicit information; SYNT, Syntactic knowledge; SUM, Summarizing ideas; GRAM, Grammar knowledge; GEN, Generalizing or synthesizing ideas; MI, Identifying main idea; INT, Making interpretation; CON, Connecting ideas; AUTH, Recognizing authors' styles; SCAN/SKIM, Scanning or skimming; PARA, Understanding paragraphs; EVA, Evaluating text; BUILD, Building a mental model; DET, Identifying supporting details.

### 4.3 RQ3: How do the similarities and differences in cognitive reading attributes assessed in young and adult readers reflect their cognitive and linguistic development?

A comparison of the cognitive reading attributes assessed in young and adult readers highlights key similarities and differences, which reflect developmental changes in cognitive and linguistic processing. Table 10 presents the distribution of attributes across the two groups, showing that while both young and adult readers rely on fundamental comprehension skills such as finding explicit information and making inferences, the complexity and scope of assessed attributes increase with age.

**Table 10:** Frequency and percentage of attributes used in assessing reading skills of adult and young readers.

**No**.	**Attributes**	**Adult readers**		**Young readers**	
		**Frequency**	**Percentage**	**Frequency**	**Percentage**
1.	Applying background knowledge	2	5%	n/a	n/a
2.	Building a mental model	3	8%	n/a	n/a
3.	Connecting ideas	5	14%	n/a	n/a
4.	Evaluating text	4	11%	5	45%
5.	Expressing in written forms	n/a	n/a	2	18%
6	Finding explicit information	29	78%	11	100%
7	Grammar knowledge	10	27%	4	36%
8	Holding memory	1	3%	n/a	n/a
9	Identifying main idea	9	24%	n/a	n/a
10	Identifying supporting details	4	11%	n/a	n/a
11	Lexical knowledge	32	86%	5	45%
12	Making inferences	31	84%	11	100%
13	Making interpretation	8	22%	6	55%
14	Negation	2	5%	n/a	n/a
15	Pragmatic knowledge	1	3%	n/a	n/a
16	Predicting	1	3%	n/a	n/a
17	Recognizing authors' styles	5	14%	1	9%
18	Scanning or skimming	5	14%	n/a	n/a
19	Sequencing	n/a	n/a	1	9%
20	Syntactic knowledge	17	46%	2	18%
21	Summarizing ideas	14	38%	3	27%
22	Synthesizing	9	24%	6	55%
23	Understanding graphs	n/a	n/a	2	18%
24	Understanding paragraphs	5	14	n/a	n/a

For young readers, the findings indicate that “finding explicit information” and “making inferences” are the most emphasized skills, with both being assessed 100% of the time. These skills are fundamental in evaluating reading comprehension, as they help determine a reader's ability to understand and extract meaning from texts. Moderately assessed skills include “synthesizing” and “making interpretations” (55%), which suggest an effort to measure higher-order thinking, along with “lexical knowledge” and “evaluating content” (45%), highlighting the role of vocabulary and critical assessment in reading. Meanwhile, “grammar knowledge” (36%) and “summarizing ideas” (27%) are given less emphasis, suggesting that structural language knowledge and text summarization are secondary to comprehension skills. Less frequently assessed attributes include “understanding graphs”, “expressing in written forms”, and “syntactic knowledge” (18%), which may reflect their lesser role in early reading development. Finally, “sequencing” and “recognizing authors' styles” are the least prioritized skills, assessed only 9% of the time. The findings suggest a strong focus on comprehension and meaning-making, with less emphasis on linguistic structure and stylistic analysis in assessing young readers' abilities.

As for adult readers, the findings reveal that “lexical knowledge” (86%), “making inferences” (81%), and “finding explicit information” (76%) are the most frequently assessed attributes in evaluating adult readers' skills. This suggests a strong emphasis on vocabulary knowledge, the ability to draw conclusions, and identifying direct information from texts. “Syntactic knowledge” (46%) and “summarizing ideas” (35%) are also significant, reflecting the importance of understanding sentence structures and condensing key points. “Grammar knowledge” (27%), along with “identifying the main idea” and “synthesizing” (24% each), highlights a moderate focus on language rules and higherorder thinking. “Making interpretation” (22%) indicates a role in deeper text analysis. Several other attributes, including “understanding paragraphs”, “scanning or skimming”, “connecting ideas”, and “recognizing authors' styles” (14% each), receive less emphasis, suggesting they are assessed selectively. “Identifying supporting details” and “evaluating content” (11% each) are considered minor components, while “building a mental model” (8%) is assessed infrequently. The least prioritized attributes include “negation” and “applying background knowledge” (5% each), as well as “predicting sequences of events”, “pragmatic knowledge”, and “holding memory” (3% each) receiving minimal attention. The findings highlight a strong focus on comprehension, vocabulary, and sentence structure in adult reading assessments, while aspects related to memory, background knowledge, and prediction are less emphasized.

**Figure 4:**
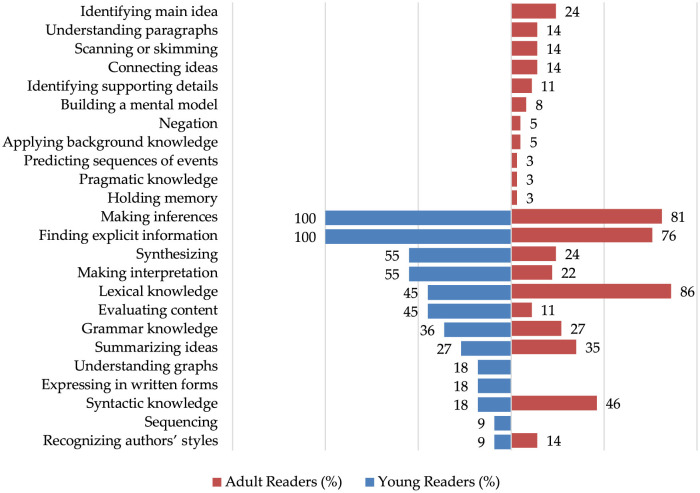
Comparison of attributes employed to assess reading skills of young and adult readers.

Moreover, studies involving adult readers have assessed a greater number of attributes, totaling 21, compared to 13 attributes identified in studies on young readers, as illustrated in Figure 4. Notably, certain attributes were exclusively examined in CDA studies on adult readers, including understanding paragraphs, connecting ideas, applying background knowledge, and pragmatic knowledge. Conversely, some attributes were uniquely assessed among young readers, such as understanding graphs and sequencing.

## 5 Discussion

### 5.1 Developmental progression of cognitive reading skills

The findings of this review indicate that reading frameworks applied across age groups are shaped by varying conceptualizations of cognitive reading processes. In young readers, the dominant attributes, finding explicit information and making inferences, suggest a framework that primarily focuses on basic comprehension and text-based understanding. These attributes align with early reading development models, which emphasize the acquisition of literal comprehension skills as the foundation for reading proficiency (Kim and Suk, 2020). The analysis reveals that young readers are predominantly assessed on literal and interpretive levels of comprehension (Nurjanah and Putri, 2020), reflecting a framework that prioritizes understanding surface-level information and basic meaning derivation. This observation also aligns with findings from neurocognitive studies, where children rely more on literal processing, supported by stronger inter-regional connectivity (Liu et al., 2018). However, the limited inclusion of syntactic knowledge, along with the absence of critical and creative comprehension skills such as evaluating content or connecting ideas, suggests that early reading frameworks place less emphasis on complex higher-order cognitive processes, which are essential for developing more sophisticated reading abilities.

Conversely, the more diverse set of attributes in adult readers' assessments highlights a broader cognitive spectrum, where vocabulary mastery, sentence structure, and higher-order skills such as synthesizing ideas and evaluating content are crucial components of reading competence. This shift signals a more holistic reading framework in adult readers, consistent with interactive and strategic reading models that require integrating both text-based and knowledge-based processes (Khalifa and Weir, 2009). The broader cognitive spectrum observed in adult readers is further supported by neurocognitive evidence, which indicates that adults not only rely on semantic processing networks (Liu et al., 2024). As reading development progresses, semantic, pragmatic, and syntactic networks play a crucial role in predicting reading ability at earlier stages of life, while more complex integrative networks emerge as stronger predictors of reading proficiency later in life (Horowitz-Kraus et al., 2024). This developmental trajectory suggests that the future-reading network, which involves the integration of multiple cognitive processes, becomes more prominent as readers mature, allowing adult readers to engage with narratives in a more sophisticated manner.

### 5.2 Diagnostic precision in measuring higher-order reading skills

The diagnostic precision of cognitive diagnostic assessment models in capturing higher-order reading skills such as synthesizing, evaluating, and interpreting texts remains a contentious issue across different age groups. While these skills are more frequently assessed in adult readers, their limited use in young readers' assessments could stem from both genuine developmental constraints and methodological limitations. Higher-order cognitive processes often require abstract reasoning, metacognitive awareness, and the integration of prior knowledge, abilities that are typically more advanced in older readers. This aligns with the hierarchical structure of reading development, where early stages focus on literal comprehension and basic meaning retrieval, while more advanced stages involve critical appraisal and inferential thinking (Chen et al., 2023). Nevertheless, the limited inclusion of higherorder skills in young readers' assessments may also be attributed to the inherent complexity of these skills and the difficulty of designing reliable, scalable tasks that isolate and measure them independently (Molokopeeva and Simard, 2024). Unlike lower-level processes, which can be assessed using controlled measures with clear indicators of specific deficits, higher-order reading involves interconnected cognitive processes that operate simultaneously (Alderson et al., 2015).

Moreover, automaticity in lower-level skills such as retrieving explicit information and lexical knowledge is essential for freeing up cognitive resources needed for more complex comprehension tasks (Suzuki and Elgort, 2023). Therefore, CDA models may prioritize lower-level attributes to establish a solid foundation before progressing to higher-order skills. However, this approach risks oversimplifying the reading process and underestimating young readers' potential to engage in advanced cognitive tasks when provided with appropriate scaffolding and instructional support. Developing ageappropriate assessments that integrate both fundamental and higher-order skills is essential to providing a more detailed diagnostic profile of young readers' cognitive abilities. Additionally, CDA models often rely on retrofitting existing assessments, which are not originally designed to probe such complex cognitive processes (Ketabi et al., 2021). Consequently, the underrepresentation of higher-order skills might not solely reflect developmental readiness but rather the inadequacy of assessment tools to elicit and diagnose these abilities. Addressing this limitation requires the development of customized assessment instruments that integrate distinct item formats, interactive tasks, and multimodal stimuli to better capture the gradual emergence of higher-order thinking in young readers (Hemati and Baghaei, 2020).

## 6 Conclusion

This study has addressed all the three research questions by critically analyzing the cognitive attributes used in reading assessments across different age groups and providing valuable insights into how reading skills are conceptualized and measured from a cognitive perspective. By comparing the attributes used in assessments of young and adult readers, the study highlights the distinct cognitive demands that readers encounter at different stages of development. The findings underscore the importance of age-specific approaches to reading instruction and assessment, which are essential for fostering reading proficiency across the lifespan. Ultimately, this research contributes to the broader discourse on educational assessment by advocating for more differentiated and contextually relevant assessment practices. By aligning reading assessments with the cognitive capabilities of learners, educators, policymakers, and assessment developers can better support the development of literacy skills to ensure that all individuals are equipped with the reading abilities necessary for their academic and professional success.

### 6.1 Theoretical and practical implications

This study contributes to the theoretical understanding of reading skill development across different age groups by highlighting the distinct cognitive attributes prioritized in assessing young and adult readers. The findings support and extend existing CDA frameworks by offering a comparative analysis of how reading skills are conceptualized and measured at various stages of life. By distinguishing between foundational skills emphasized in young learners and more complex, abstract skills in adults, the study provides a comprehensive perspective on the cognitive processes underlying reading proficiency. This reinforces the need for age-specific theoretical models that account for the evolving nature of reading comprehension and the adaptive strategies employed by readers as they mature.

The review also suggested that internal and external stakeholders should adjust their approaches based on the findings. Concerning internal stakeholders, especially educators, who play a key role in decision-making that directly impacts the institution's operations and pedagogical direction (Chan and Chou, 2020), the findings have direct implications for the design and implementation of reading instructional strategies and assessments. The identification of key reading attributes for different age groups enables educators to tailor their teaching methods and assessment tools to better align with the cognitive abilities of their students. For young learners, the emphasis on attributes like making inferences and finding explicit information suggests that teaching strategies should focus on enhancing these critical interpretive skills. In contrast, for adult learners, greater attention should be paid to vocabulary development, syntactic knowledge, and summarization techniques. Although readers of all ages must develop a range of reading skills, young learners acquire language implicitly through exposure, building interpretive abilities like analysis and inference that are essential for academic success. In contrast, adult learners benefit from explicit instruction in vocabulary, syntax, and summarization, which are vital for comprehending more complex texts and engaging in professional or academic reading. Thus, these distinctions highlight the need for a differentiated instructional approach that accommodates the developmental and cognitive differences between young and adult learners.

Regarding external stakeholders, such as policymakers, curriculum developers, educational authorities, and assessment developers, who influence decisions, shape policies, and establish frameworks impacting the institution's operations within the broader educational ecosystem (Agnew, 2021), the study highlights the need for reading assessments that accurately reflect the cognitive demands placed on readers of different ages. The findings advocate for a more differentiated approach to assessment design, one that recognizes the varying complexities of reading tasks faced by young and adult readers. This has implications for standardized testing and literacy programs, suggesting that a one-size-fits-all model is inadequate. External stakeholders are encouraged to consider these insights when formulating educational policies, designing literacy interventions, and developing new assessment tools that are both equitable and effective across diverse populations.

### 6.2 Limitations and recommendations

Despite offering valuable insights, this study acknowledges several limitations that highlight opportunities for further research. The study focuses on reading assessment research through the cognitive diagnostic approach, which provides a fine-grained understanding of reading attributes but may not fully capture perspectives from other emerging assessment frameworks. The use of the scoping review methodology in identifying, screening, and analyzing publications facilitates a comprehensive mapping of the literature, yet it does not offer the quantitative synthesis that could be achieved through meta-analyses. Additionally, the study is confined to English language resources and reading assessments in the context of English as a first, second, or foreign language, which may restrict the generalizability of the findings across diverse linguistic and cultural settings. The selection of studies from three reputable academic databases with a focus on empirical studies ensures the inclusion of high-quality publications but may exclude valuable insights from other sources such as conference proceedings, book chapters, and gray literature.

To address these limitations, future research could explore several key areas. Future reviews on advanced technologies in reading assessment, such as eye tracking and electroencephalography (EEG), are needed to provide objective, real-time insights into readers' cognitive processes and offer a more holistic understanding of reading behaviors. Additionally, future studies could undertake more rigorous review methodologies, such as meta-analyses and bibliometric studies, to provide a more extensive understanding of prevailing trends and gaps in the literature on cognitive reading attributes. Cross-linguistic comparative research would also be valuable in examining the consistency of cognitive reading attributes across different languages and cultural contexts, thereby enhancing the generalizability of the findings. Furthermore, comparative investigations between conventional and technology-assisted assessments could yield critical insights into the effectiveness, adaptability, and efficiency of different assessment approaches. Finally, evaluating the strengths and limitations of various reading assessment methods would help identify the most reliable and valid techniques for measuring reading proficiency across diverse age groups and educational settings. Addressing these avenues would significantly enrich the understanding of cognitive reading attributes and contribute to the advancement of reading assessment practices in various linguistic and educational contexts.

## Data Availability

The original contributions presented in the study are included in the article/supplementary material, further inquiries can be directed to the corresponding author.
